# Quantitative *operando* visualization of the energy band depth profile in solar cells

**DOI:** 10.1038/ncomms8745

**Published:** 2015-07-13

**Authors:** Qi Chen, Lin Mao, Yaowen Li, Tao Kong, Na Wu, Changqi Ma, Sai Bai, Yizheng Jin, Dan Wu, Wei Lu, Bing Wang, Liwei Chen

**Affiliations:** 1 i-Lab, Suzhou Institute of Nano-Tech and Nano-Bionics, Chinese Academy of Sciences, Suzhou 215123, China; 2 Hefei National Laboratory for Physical Sciences at the Microscale and Synergetic Innovation Center of Quantum Information & Quantum Physics, University of Science and Technology of China, Hefei 230026, China; 3 Advanced Opto-electronic Materials Lab of Soochow University, Department of Polymer Science and Engineering, College of Chemistry, Chemical Engineering and Materials Science, Soochow University, Suzhou 215123, China; 4 Division of Nanobiomedicine, Suzhou Institute of Nano-Tech and Nano-Bionics, Chinese Academy of Sciences, Suzhou 215123, China; 5 Division of Printed Electronics, Suzhou Institute of Nano-Tech and Nano-Bionics, Chinese Academy of Sciences, Suzhou 215123, China; 6 State Key Laboratory of Silicon Materials, Department of Materials Science and Engineering, Zhejiang University, Hangzhou 310027, China

## Abstract

The energy band alignment in solar cell devices is critically important because it largely governs elementary photovoltaic processes, such as the generation, separation, transport, recombination and collection of charge carriers. Despite the expenditure of considerable effort, the measurement of energy band depth profiles across multiple layers has been extremely challenging, especially for *operando* devices. Here we present direct visualization of the surface potential depth profile over the cross-sections of *operando* organic photovoltaic devices using scanning Kelvin probe microscopy. The convolution effect due to finite tip size and cantilever beam crosstalk has previously prohibited quantitative interpretation of scanning Kelvin probe microscopy-measured surface potential depth profiles. We develop a bias voltage-compensation method to address this critical problem and obtain quantitatively accurate measurements of the open-circuit voltage, built-in potential and electrode potential difference.

## Abstract (web summary)

The energy band alignment of solar cell materials is highly relevant to the device performance, but its measurement is challenging. Here, the authors report direct visualization of energy band alignment in operating organic photovoltaic devices using scanning Kelvin probe microscopy imaging.

## Graphical abstract

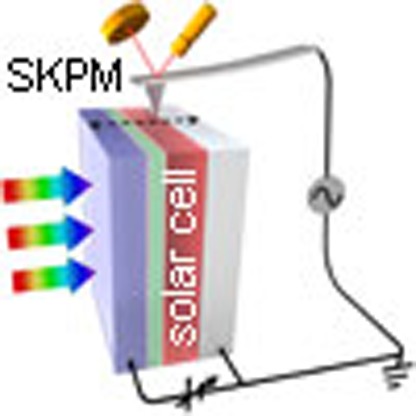

The energy band alignment across multiple layers is extremely important for solar cells because elementary photovoltaic processes, such as charge separation, carrier transport and collection^1^,^2^,^3^,^4^, as well as undesirable recombination^5^,^6^, all depend on this alignment. However, experimental determination of the energy-level alignment and the quantitative measurement of related parameters, such as the built-in potential (*V*_bi_), have been difficult to realize, especially under typical operating conditions for the devices. Indirect methods, such as Mott–Schottky analysis^7^,^8^,^9^,^10^, electroabsorption spectroscopy^11^,^12^,^13^ and dark current density–voltage *(J–V*) curve fitting^9^,^14^, have been applied to measure the *V*_bi_ in solar cells. Scanning Kelvin probe microscopy (SKPM), a functional imaging technique that maps the local surface potential (SP) of a sample^15^,^16^, has also been employed to measure the potential difference between adjacent layers in solar cells^17^,^18^,^19^,^20^.

Recently, SKPM has been used to image cross-sections of solar cells to probe the SP depth profile across multiple layers^21^,^22^,^23^,^24^,^25^,^26^. The vacuum level (VL) in an energy band structure is defined as the energy of an electron resting right at the surface of a material; thus, the VL alignment within a device can be obtained by multiplying the SP depth profile by a constant, that is, the electron charge^27^,^28^. In principle, this approach offers the advantage of being able to directly visualize the energy band alignment across the device. However, a major obstacle has limited the application of this approach in solar cell research: quantitatively accurate measurements and interpretations are yet to be realized in cross-sectional SKPM studies. Critical parameters for the operating mechanism of solar cells, such as the open-circuit voltage (*V*_oc_) and *V*_bi_, are often found to be much smaller than expected from *J–V* characterization^21^,^22^,^23^. Overall, the results obtained from cross-sectional SKPM measurements are often uncorrelated with the device performance and *J–V* characteristics, and are thus not applicable for understanding practical devices in their actual operating states.

In this study, we address the challenges in quantitative SKPM potentiometry using high-efficiency organic photovoltaic (OPV) cells^29^,^30^,^31^ as model systems. As shown in Fig. 1a, cross-sections of OPV devices are exposed by using ion-beam milling, and the SP depth profiles of the OPV device under operating conditions are directly visualized using SKPM imaging on the cross-section (Fig. 1b). The tip/cantilever convolution effect due to finite tip size and cantilever beam crosstalk is identified as the source of the systematic underestimate of SP differences in cross-sectional SKPM measurements^32^,^33^,^34^. A bias compensation method is developed to obtain quantitative measurements of internal potential difference parameters, such as *V*_oc_, electrode potential difference (*V*_td_) and *V*_bi_, in OPV devices.

## Results

### Preparation of OPV device cross-sections

**Figure 1:**
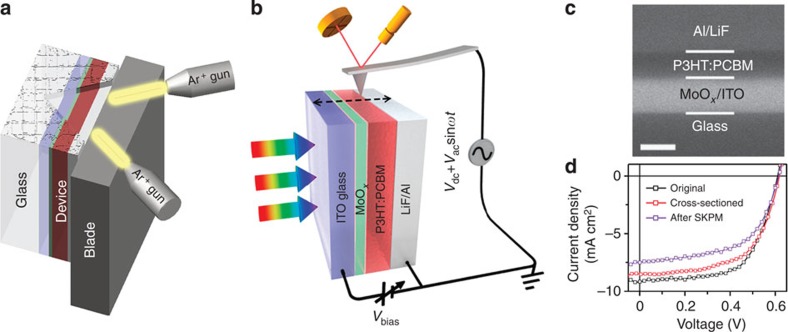
*Operando* **probing of solar cell energy band depth profiles with cross-sectional SKPM.** (**a**) Schematic illustration of ion-beam-milling configuration to expose a smooth cross-section of an OPV device; (**b**) schematic illustration of cross-sectional SKPM measurements under operating conditions such as illumination and bias voltages. The cantilever approaches the sample from the side opened up using ion-beam milling. SKPM scanning is carried out with the cantilever long axis normal to the device plane; (**c**) SEM micrograph of an ion-beam-milled OPV device cross-section (scale bar, 250 nm); and (**d**) current density versus voltage (*J–V*) characteristics of an OPV device with a conventional configuration ITO/MoO_*x*_/P3HT:PCBM/LiF/Al under AM 1.5G illumination before (black) and after (red) cross-section preparation, and after SKPM measurement (voilet), indicating good device stability.

The OPV devices are fabricated on the glass substrate using a conventional stacking of indium tin oxide (ITO)/MoO_*x*_/active materials/LiF/Al. To prepare a smooth cross-section, the device was cut from the back of the glass substrate. The exposed edge is then milled under an Ar^+^ beam (Ilion^+^ 693, Gatan Inc., Pleasanton, CA, USA; Fig. 1a). The device fabrication and cross-section preparation are described in details in the Methods section. Figure 1c displays the scanning electron microscopy (SEM)-backscattered electron image of the cross-section. The contrast in the image primarily results from materials with different atomic numbers rather than the topography. The cross-section morphology is further confirmed using X-ray energy dispersive spectrum mapping in Supplementary Fig. 1. *J–V* curves of a typical bulk heterojunction (BHJ) device that is obtained using poly(3-hexylthiophene) (P3HT):[6,6]-phenyl-C_61_-butyric acid methyl ester (PCBM)-active materials exhibit a power conversion efficiency (PCE) of 3.47% (Fig. 1d, Table 1). The same device exhibits PCE of 3.17% after cross-section ion milling and 2.65% after SKPM imaging with very stable *V*_oc_. Cross-sectioning was shown to result in small drops in *J*_sc_ (∼6.98% drop) and fill factor (FF; ∼3.23% drop), while the SKPM imaging resulted in further decreases in *J*_sc_ (∼11.23% drop) and FF (∼4.84% drop). Although the chemical changes associated with these performance decreases may, in principle, be significant, we believe that the cross-sectional SP mapping retains useful qualitative information about the pristine devices because the device *V*_oc_ is well maintained throughout the cross-sectioning and measurement processes. Since the device is exposed to the ambient environment during the ion-beam milling, the device stability under ambient condition and the conditions of SKPM measurements is highly important. Note that, in this study, it is critical to replace the typical poly(3,4-ethylenedioxythiophene):poly(styrenesulfonic acid) (PEDOT:PSS) anode interlayer with MoO_*x*_ because PEDOT:PSS is detrimental to the device stability^35^. Supplementary Fig. 2 shows that this device maintained ≃68% of its original PCE after 100 h of exposure to the ambient environment, and the drop in efficiency over the first 40 h is only ∼2%. In contrast, the devices with PEDOT:PSS interlayers decay much faster and completely lose their photovoltaic properties in ≃80 h. The successful preparation of the cross-section and maintenance of device stability during SKPM measurements enable us to investigate the energy band profiles in *operando* devices.

### Qualitative energy band alignment in *operando* BHJ devices

**Figure 2:**
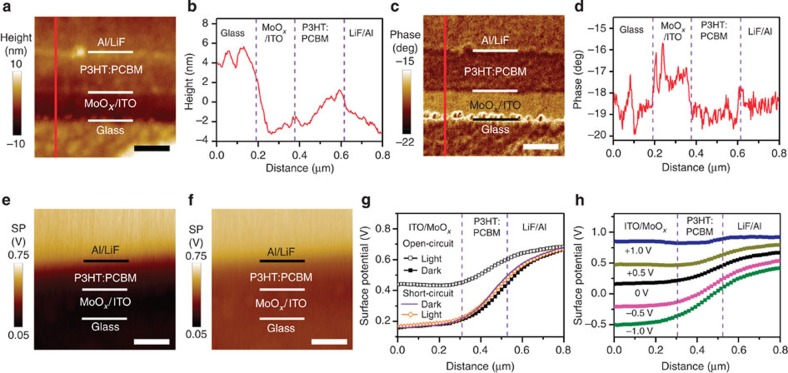
Cross-sectional images and depth profiles of a BHJ device. (**a**) Topography and (**c**) phase images of the ITO/MoO_*x*_/P3HT:PCBM/LiF/Al device cross-section obtained with AFM (scale bars, 200 nm). The line profiles in **b**,**d** correspond to the red lines in **a**,**c**, respectively. SP images of the P3HT:PCBM BHJ device in open-circuit (**e**) in the dark and (**f**) under AM 1.5G illumination (scale bar, 250 nm); (**g**) SP depth profiles of the device in open-circuit in the dark (solid black squares), in open-circuit under AM 1.5 G illumination (open black squares), in short-circuit in the dark (solid purple line) and in short-circuit under AM 1.5 G illumination (open orange diamonds); (**h**) SP depth profiles of the P3HT:PCBM BHJ device in the dark with bias voltages ranging from −1.0 to +1.0 V. The wiring configuration for applying the bias voltage is illustrated in Fig. 1b.

Figure 2a,c is topographical and phase images, respectively, of the cross-sections that are obtained via atomic force microscopy (AFM). The morphology of the ion-milled cross-section is relatively smooth with an ≃10-nm-height difference between layers (Fig. 2b). A sharp contrast in the phase channel (Fig. 2d) is resulted from different mechanical properties of the organic and inorganic layers and is used to identify the interfaces within the device.

**Figure 3:**
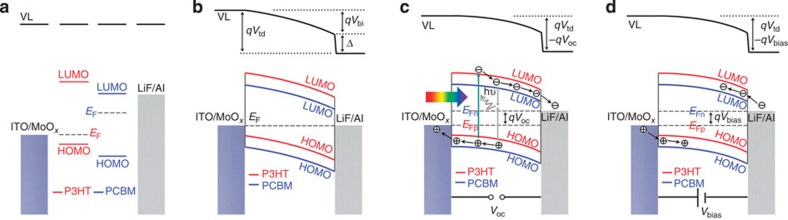
Device energy band diagram under different conditions. (**a**) VL alignment of an ITO/MoO_*x*_/P3HT:PCBM/LiF/Al BHJ device before contact; (**b**) Fermi-level alignment after contact in the dark; (**c**) separated quasi-Fermi levels for electrons and holes under illumination; and (**d**) separated quasi-Fermi levels due to bias voltages between the two electrodes. Here, HOMO means highest occupied molecular orbital and LUMO means lowest unoccupied molecular orbital.

In equilibrium states, the Fermi levels are aligned across any interfaces. In OPV devices, the difference in the work function of the materials leads to VL offsets across different layers. However, the physical and chemical interactions between different materials in contact with each other may result in the formation of interfacial electronic states, interfacial charge transfer and the occupation of interfacial states, producing complicated interfacial dipole moments and band-bending^7^,^9^,^20^,^27^,^28^,^36^. Thus, energy band alignment in devices is difficult to predict and has not been quantitatively measured in experiments so far. Figure 2e,f presents SP images of the device cross-section acquired with an amplitude-modulation (AM) mode SKPM under open-circuit conditions in the dark and under AM 1.5 G illumination, respectively. The SP depth profile across the device (Fig. 2g) is obtained by averaging at least 10 scan lines in the slow-scan direction. The SP profile under short-circuit conditions in the dark is also shown and essentially overlaps with the open-circuit SP profile. The depth profiles in Fig. 2g show two important qualitative features: first, the potential drop is distributed throughout the entire active layer and, second, the SP decreases more rapidly at the cathodic (LiF/Al) interface than at the anodic (MoO_*x*_/ITO) interface (confirmed by the derivative of the SP profile as shown in Supplementary Fig. 3). These results can be explained using the energy band diagrams that are shown in Fig. 3. As the different layers (Fig. 3a) are fully in contact within the device, Fermi-level alignment is established irrespective of whether the configuration is a short-circuit or an open-circuit in the dark. The difference in the material work function is then reflected in the variation in the VL across the layers (Fig. 3b). It has been reported that P3HT is enriched at the anodic interface because of vertical phase segregation within the BHJ^37^,^38^. The work function of P3HT is very close to that of the anode; thus, the VL shift at the anodic interface is negligible, and the change in the SP at the anodic side is rather slow. At the cathodic side, the work function offset between LiF/Al and the P3HT:PCBM mixture is compensated for by an interface state-induced dipole moment and band bending within the active layer to achieve the Fermi-level alignment. The interface states may arise from physical and chemical interactions between the organic material and the metal electrode^7^. The low work function of LiF/Al can result in the injection of electrons from the metal electrode to the interface states. These localized charged states form an electrical dipole moment that causes a sharp VL shift at the cathodic interface, *Δ*. However, the density of interface states in organic materials is typically much lower than that in inorganic semiconductors, and the corresponding interfacial dipole is not sufficiently strong to fully compensate for the work function difference. Therefore, the LiF/Al electrode injects more electrons into the P3HT:PCBM BHJ, resulting in band bending (*qV*_bi_) in the active layer. This picture explains the continuous potential drop across the entire active layer and the rapid potential change at the cathode interface. Overall, the VL shift at the cathode interface (*Δ*) and the band bending (q*V*_bi_) in the active layer add up to the total energy drop from the anode to the cathode (*qV*_td_; Fig. 3b).

The SP profile exhibits considerable differences under illumination in the open-circuit configuration (Fig. 2g). The total SP drop from the cathode to the anode, *V*_td_, is reduced under illumination in the open-circuit configuration. This result is obtained because of the accumulation of photo-generated carriers at the two electrodes, which establishes the quasi-Fermi levels of the hole (*E*_Fp_) and the electron (*E*_Fn_) at the anode and cathode, respectively. The direction of the field generated by the accumulated photo-generated carriers is opposite to that of the built-in field; therefore, the overall VL drop across the device under open-circuit conditions and illumination is less than that for the corresponding case in the dark (Fig. 3c).

The observed SP profile of the *operando* device, which is optimized with a P3HT:PCBM-active layer thickness of ≃200 nm, is consistent with the device performance. The continuous potential drop indicates the existence of a built-in electric field across the entire BHJ. The large donor–acceptor interface area in the bi-continuous BHJ facilitates the dissociation of photo-generated excitons into geminate carrier pairs; the built-in field further separates the geminate pairs and prevents geminate recombination^5^,^6^, resulting in a high short-circuit current density (*J*_sc_) and a high FF of >0.6 in the device.

### Quantitative energy-level offsets in *operando* BHJ devices

To further diagnose the effects of the materials and the device configuration on the device performance, it is extremely important to obtain quantitative information on the energy band alignment. However, careful analysis reveals that the energy-level offsets directly obtained from the data shown in Fig. 2g are smaller than that expected from the design and performance of the device. For example, the reduction in *V*_td_ in an open-circuit configuration under illumination from that in the dark state should be equal to the device *V*_oc_ (0.61 V); however, a much smaller value (0.25 V) is found in the SKPM measurement. Furthermore, the energy band diagram in Fig. 3 shows that the *V*_td_ in the dark state should be equal to the difference in the work function of the electrodes of 1.6 V (the work functions of the MoO_*x*_/ITO and LiF/Al electrodes have been separately measured to be 5.3 and 3.7 eV using ultraviolet photon spectroscopy (UPS) and a dark *J–V* fitting method on the basis of a diode model, respectively)^14^,^39^. However, the SKPM-measured value is 0.50 V (Fig. 2g). This discrepancy in the quantitative values has previously been encountered in studies on device cross-sections^21^,^22^,^23^ and has become a major obstacle in analysing device performance using the cross-sectional SKPM potentiometry.

**Figure 4:**
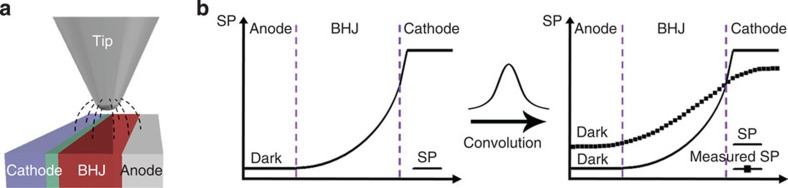
The tip-induced averaging effect in SKPM measurements. (**a**) Schematic illustration of a finite-sized SKPM tip scanning over a cross-section of a multilayered thin-film device; (**b**) an illustration of the measured SP profile resulted from the convolution of the true profile with the transfer function of the SKPM tip.

We suspect that these results originate from systematic artefacts in SKPM including the finite-size tip convolution effect and the cantilever beam crosstalk. SKPM exploits long-range electrostatic forces; therefore, interaction between a large area of the sample and the entire tip/cantilever assembly (including the tip apex, the cone- or pyramidal-shaped tip and the cantilever beam) is probed (Fig. 4a)^34^,^40^. These effects can be mathematically treated with the convolution of the true potential profile with a transfer function, which is dependent on the tip size, shape, tip–sample distance and so on, and is often modelled with a Gaussian function (Fig. 4b)^32^,^41^. If the width of the transfer function is much smaller than the sample feature size, the artefacts can be relatively minor (Supplementary Fig. 4); however, when the sample feature size is comparable to the tip size and tip–sample distance, the effects of tip/cantilever convolution may cause an apparent smoothing and averaging in measured profiles (Fig. 4b)^42^. Different methods such as the frequency-modulation mode SKPM^32^ and scan configuration optimization^33^ have been explored to reduce the artefacts in SKPM measurements; however, the effect of finite tip size and cantilever crosstalk cannot be completely eliminated. Since the cross-section of OPV devices is composed of multiple layers of different materials with a total thickness of ∼500 nm (the active layer is 200-nm thick), which is comparable to the tip size (∼20 nm in the tip apex radius and ∼10 μm in tip height) and the average tip–sample distance in experiments (typically 10-nm lift height plus 20-nm tip oscillation amplitude), it is highly plausible that the tip/cantilever convolution effects may drastically influence the SKPM potentiometry in our experiments.

The SP profiles of the device under different bias voltages (*V*_bias_) between the anode and cathode (Fig. 2h) further corroborate this hypothesis. In the experimental configuration shown in Fig. 1b, a forward *V*_bias_ introduces an electric field in the same direction as the photo-induced electric field, which separates the quasi-Fermi levels and decreases *qV*_td_ (Fig. 3d); vice versa, a reverse *V*_bias_ enlarges the total potential drop. Ideally, the *V*_td_ should decrease by 1.0 V under a forward *V*_bias_ of +1.0 V; however, the apparent reduction in *V*_td_ is only 0.41 V, as shown in Fig. 2h. Similarly, the *V*_td_ should increase by 1.0 V under a reverse *V*_bias_ of −1.0 V, but an increase in only 0.40 V is seen in Fig. 2h. The variation in *V*_td_ is plotted against *V*_bias_ in Supplementary Fig. 5. A good linear correlation is found for the curve; however, the slope is considerably less than 1 (≃0.41), confirming the existence of a systematic and severe underestimate of SP differences in these measurements.

**Figure 5:**
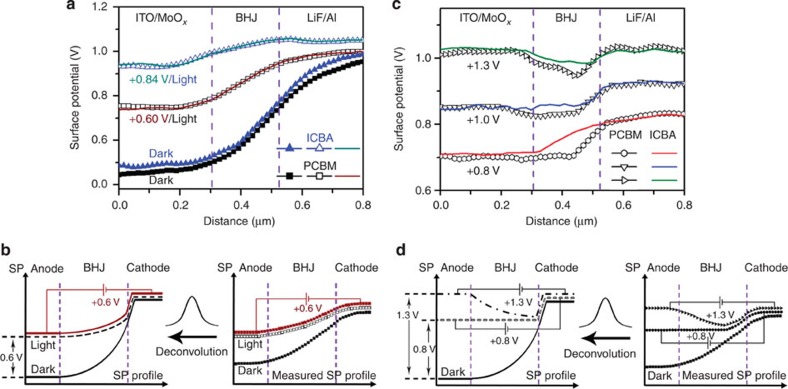
Quantification of energy-level offsets by using the bias compensation method. (**a**) SP depth profiles of ITO/MoO_*x*_/P3HT:acceptor/LiF/Al devices with PCBM or ICBA acceptors in the dark, under AM 1.5 G illumination and at biased conditions (0.60 and 0.84 V forward bias on the PCBM and ICBA devices, respectively). (**b**) Illustration of the quantitative measurement of *V*_oc_ using the data from P3HT:PCBM device as an example. Overlapping SP profiles in SKPM measurements means that the actual profiles are also identical if they had been deconvoluted. (**c**) SP depth profiles of the two devices in the dark with different bias voltages of +0.8, +1.0 and +1.3 V. (**d**) Illustration of the bias compensation method using the data from the P3HT:PCBM device as an example. Flat-band and levelled electrode conditions would remain if the measured profiles could be deconvoluted.

Interestingly, a quantitative correlation is observed between the *J–V* performance of the OPV device and SKPM measurements. The SP profile obtained under a forward *V*_bias_ of +0.60 V appears to be identical to the SP profiles in open-circuit under illumination (Fig. 5a, the original SP images are shown in Supplementary Fig. 6). The overlay of the two measured profiles means that the actual profiles would also be identical if they had been deconvoluted (Fig. 5b). That is to say, the *V*_oc_ measured from SKPM is 0.6 V, which is well agreed with the 0.61 V from device *J–V* measurements.

It is not a simple coincidence that a bias voltage equal to *V*_oc_ is required to overlay the SP profile in the dark to that under illumination. The same result is observed for P3HT:indene-C_60_ bisadduct (ICBA) BHJ devices (Fig. 5a), which have the same device structure as the aforementioned P3HT:PCBM BHJ devices except that a different acceptor material is used, ICBA. The fabrication of the P3HT:ICBA device is described in detail in the Methods section. Figure 5a shows that the SP profiles of PCBM and ICBA devices are almost identical in the dark, but that the *V*_td_ is reduced by 0.38 V for the ICBA device on illumination, which is considerably larger than the corresponding reduction of 0.25 V for the PCBM device. A *V*_bias_ of +0.84 V is required to overlay the SP profile with that of the P3HT:ICBA device under illumination (the original SP images are shown in Supplementary Fig. 7), which is equal to the *V*_oc_ that is obtained from the *J–V* characteristics (Supplementary Fig. 8). The higher *V*_oc_ value for the ICBA device compared with that of the PCBM device is in good agreement with reports in the literature (detailed parameters of the ICBA device before and after cross-section preparation and after SKPM measurement are in Supplementary Fig. 9 and Table 1)^43^.

The accurate measurement of *V*_oc_ indicates that SKPM potentiometry can yield quantitative information in spite of the presence of severe tip/cantilever convolution effect. The external bias between the anode and cathode plays an important role in this process and can be further exploited for the quantitative measurements of other energy-level offsets in device depth profiles. For example, the measured profile in Fig. 2g is strongly influenced by the convolution effect: the potential value along the line is smoothed and averaged, leading to an underestimate of potential differences between points along the profile. To obtain the true SP difference between two points, for example, the cathode and the anode, we may apply an external bias between the electrodes and look for the bias voltage required to equalize the SP of the two electrodes. When the SP difference is fully compensated by the external bias, the two electrodes are at the same potential. At this point, the effect of smoothing and averaging due to tip/cantilever convolution is minimized, and the external bias voltage reflects the true SP difference (Fig. 5d). It is emphasized that this bias compensation method does not reduce or remove the tip/cantilever convolution effect, but, instead, this method seeks to create an equal potential condition where the effect of convolution is minimized, such that the true SP difference between certain energy levels can be probed.

Figure 5c shows the measurement of *V*_td_ in the P3HT:PCBM and P3HT:ICBA BHJ devices using the bias compensation method. *V*_td_ is an important parameter in the energy band alignment. As explained in Fig. 3b, the value of *qV*_td_ equals the sum of *qV*_bi_ and the cathode interface VL shift (*Δ*); thus, *V*_td_ is taken to be the upper limit of *V*_bi_. In principle, q*V*_td_ can be obtained by separate measurements of the work function of the two electrodes using UPS; however, the low work function cathode material is often partially oxidized^44^, and thus the *V*_td_ has not been measured previously in operating OPV devices.

**Figure 6:**
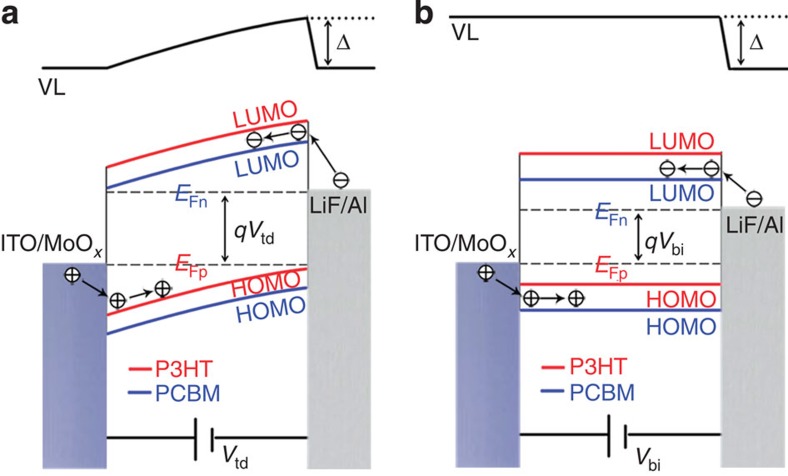
Energy band diagrams under different bias conditions. A ITO/MoO_*x*_/P3HT:PCBM/LiF/Al device under (**a**) cathode–anode VL alignment condition and (**b**) active layer flat-band condition.

In the measurement of *V*_td_ via the bias compensation method, the external bias necessary to equalize the SP of the two electrodes is searched. Since the experiments in Fig. 2h have established a monotonic correlation between the applied bias and the reduction in SP difference between the electrodes, it is thus natural to further raise the bias to equalize the electrode potentials. Figure 5c shows that when the forward *V*_bias_ is increased to +1.3 V, both the PCBM and ICBA devices show equal SPs between the cathode and the anode. This result means that the work function difference between the two electrodes is compensated by the 1.3 V forward *V*_bias_. It confirms that the overall *qV*_td_ is determined by the electrode materials and is independent of the active material. The corresponding energy band diagram of the cathode–anode VL alignment condition is shown in Fig. 6a. In addition, under a *V*_bias_ of 1.3 V, the active layer in PCBM shows higher reversed band bending and a larger SP difference between the cathode and the active layer than the ICBA, which is self-consistent with the cathode interfacial dipole of the ICBA being stronger than that of the PCBM. The difference between the work function for the MoO_*x*_/ITO and LiF/Al electrodes has never been directly measured in *operando* devices. The measured value of 1.3 eV using the bias compensation method is reasonable and fairly close to the value of 1.6 V derived from the difference between the work function of MoO_*x*_/ITO and LiF/Al electrodes, as measured by UPS and the diode-model-based dark *J–V* fitting method, respectively^14^,^39^.

Very importantly, the *V*_bi_ of BHJ OPV devices can also be measured using the bias compensation method in SKPM. Accurate measurement of *V*_bi_ is critical for understanding the device mechanism because *V*_bi_ strongly affects charge separation, transport, collection and recombination and is the upper limit of the device *V*_oc_ (refs 14, 45). In p–n junction diodes, the *V*_bi_ is defined as the potential difference across the two ends of the p–n junction, but the *V*_bi_ in a BHJ OPV is difficult to define and measure because the diode junction contains a phase-separated bi-continuous mixture. If we apply the classic metal–insulator–metal model to the OPV, the value of *qV*_bi_ equals the difference in the work function between the anode and the cathode, which manifests as *qV*_td_ in the *operando* device^45^. However, this approximation for *V*_bi_ completely neglects molecular details and interfacial states, and thus cannot accurately account for the behaviour of the device.

If we define the *V*_bi_ in an OPV similarly to that in p–n junction diodes, that is, as the potential difference across the two ends of the BHJ, the *V*_bi_ is practically equal to the *V*_bias_ that is required to level the potential of the two ends of the BHJ, that is, the flat-band potential *V*_fb_, in light of the bias compensation method (the corresponding energy band diagram is illustrated in Fig. 6b). Figure 5c shows the measurement of *V*_fb_ or *V*_bi_ of the PCBM and ICBA devices. Under a +0.8 V forward *V*_bias_, the SP profile from the anode to the most part of the active layer in the PCBM device is flat, and the only potential drop occurs at the cathodic interface (because of the interfacial dipole)^7^. Thus, we infer that the *V*_bi_ of the P3HT:PCBM device is ≃0.8 V. Taking the active layer thickness to be ≃200 nm, the built-in field is estimated to be ∼4 × 10^4 ^V cm^−1^, which falls within the ≃1–10 × 10^4^ V cm^−1^ range that was estimated in a previous publication^19^ and is close to the 8 × 10^4^ V cm^−1^ value that was obtained in a numerical simulation on the basis of the Blom model^46^ and the Nelson model^47^. In contrast, the ICBA device under +0.8 V *V*_bias_ still exhibits a continuous potential drop and significant band bending. A *V*_bias_ of +1.0 V is required to reach the flat-band condition in the ICBA device; therefore, the *V*_bi_ for the P3HT:ICBA device is ≃1.0 V. Higher *V*_bi_ in the ICBA device than that in the PCBM device is consistent with Mott–Schottky analysis (Supplementary Fig. 10). Both the 0.8 V value in the PCBM device and the 1.0 V value in the ICBA device obtained via the bias compensation method are greater than the device *V*_oc_, which is consistent with concept that *V*_bi_ is the upper limit of *V*_oc_, and therefore shall be greater than it^14^,^45^.

Thus, we have demonstrated that important parameters such as *V*_oc_, *V*_td_ and *V*_bi,_ can be quantitatively measured using the bias compensation method in spite of the presence of the tip/cantilever convolution effect in SKPM. This breakthrough is generally valuable for SKPM and particularly intriguing for mechanistic studies on OPVs and other thin-film devices.

### Energy band alignment in planar junction devices

**Figure 7:**
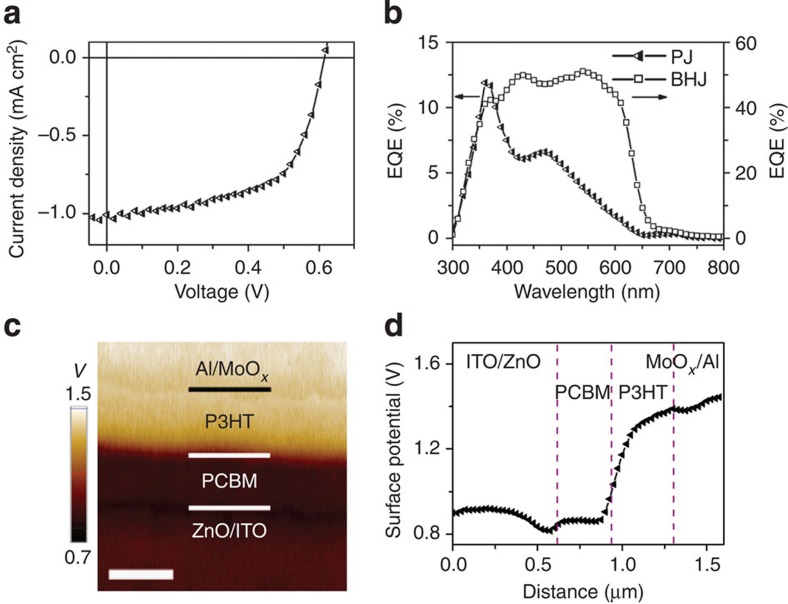
Device characteristics and SP of a PJ device. (**a**) *J–V* curve of a ITO/ZnO/PCBM/P3HT/MoO_*x*_/Al PJ device under AM 1.5G illumination, (**b**) EQE of the PJ device (triangles) and the BHJ device (squares) that are shown in Fig. 2, (**c**) SP image (scale bar, 400 nm) and (**d**) SP profile of the PJ device cross-section under short-circuit conditions in the dark.

To provide another example of the correlation between the SP profile and the device performance, we investigate a planar junction (PJ) device using P3HT- and PCBM-active materials. The preparation of the PJ device is described in detail in the Methods section. It is critical to avoid thermal annealing to minimize interlayer diffusion and obtain an abrupt donor–acceptor junction/interface between the P3HT and the PCBM^48^. Figure 7a shows the *J–V* characteristics of the ITO/ZnO/PCBM/P3HT/MoO_*x*_/Al PJ device. The *V*_oc_ (0.61 V) and FF (0.61) of the PJ device are comparable to those of the BHJ device in Fig. 1; however, the *J*_sc_ (1.01 mA cm^−2^) is much lower than that of the BHJ device. The PJ device also shows much lower external quantum efficiency (EQE) than the BHJ device (Fig. 7b), and the peak in EQE does not overlap with the absorption of active materials but severely leans towards the short-wavelength region. The cross-sectional SP image and the depth profile of the PJ device under short-circuit conditions in the dark (Fig. 7c,d) show that the majority of potential drop occurs at the interface between the P3HT and the PCBM and that there are regions in the P3HT and PCBM layers where the potential change is rather small and the electric field is very weak, which is very different from the BHJ case for which a continuous potential drop is observed across the entire BHJ layer. The fact that the VL does not align at the organic/organic, that is, P3HT/PCBM, interface is consistent with the UPS results^49^. Spontaneous electron transfer has been reported from the donor P3HT to the acceptor PCBM, which results in an interface dipole with its positive end towards the P3HT layer and its negative end towards the PCBM layer^50^. The SP depth profile in Fig. 7d is in qualitative agreement with this picture.

## Discussion

This contribution shows the realization of *operando* mapping of the energy band depth profile in OPV devices. A bias compensation method has been devised to measure energy-level offsets. This study clearly indicates that the tip/cantilever convolution effect and the associated low spatial resolution in SKPM constitute major bottlenecks in unveiling the true energy band alignment profile. Ideally, deconvoluted energy profiles with nanometre or subnanometre spatial resolution could lead to accurate calculation of the device internal field and spatially resolved understanding of the drift current within the device. While technical advances such as frequency modulation-SKPM and/or optimized scan configuration may improve the spatial resolution on the current study, ultimately, future efforts in reducing the tip size and hence actual reduction in the tip convolution effects will be highly desirable for mechanistic understanding of thin-film devices.

In conclusion, we have directly visualized energy band alignment in operating OPV devices using cross-sectional SKPM imaging. A bias compensation method allows for quantitative measurements of energy-level differences such as *V*_oc_, *V*_bi_ and *V*_td_ in operating OPV devices. The resulting SP profiles of BHJ and PJ P3HT:PCBM devices show that a continuous potential drop across the entire BHJ-active layer is critical to facilitate carrier separation and collection for high *J*_sc_ and FF in device performance, whereas the donor–acceptor interface dipole efficiently promotes the dissociation of photo-generated excitons. This approach should be generally applicable to other thin-film devices, such as perovskite and quantum dot solar cells, light-emitting diodes and thin-film transistors, and could contribute significantly to the understanding of the mechanisms in and improvements of these devices.

## Methods

### Materials

All of the active layer materials are purchased and used as received. The P3HT is obtained from 1-Material Inc., the PCBM is obtained from American Dye Source Inc. and the ICBA is obtained from Solenne BV.

### BHJ device preparation

The BHJ device is fabricated by sequentially cleaning pre-patterned ITO glass substrates (15 Ω sq^−1^) using ultrasonication for 10 min in a detergent, deionized water, ethanol, acetone and isopropyl alcohol, followed by treatment with oxygen plasma for 10 min. Then, a 10-nm MoO_*x*_ layer, which serves as an anode interlayer, is thermally evaporated on ITO substrates under a pressure of 4 × 10^−4 ^Pa. The active layer consists of different blends that are prepared by spin-coating, followed by being subjected to different annealing conditions. A 1-nm LiF layer and a 100-nm Al layer are subsequently evaporated under a pressure of 4 × 10^−4 ^Pa through a shadow mask to define the active area of the devices (0.12 cm^2^) and to form a top cathode. P3HT:PCBM (36 mg ml^−1^, 1:0.8 w/w) and P3HT:ICBA (34 mg ml^−1^, 1:1 w/w) are dissolved in 1,2-dichlorobenzene. Both blends are stirred at 60 °C for ≃14 h in a glove box. P3HT:PCBM is spin-coated at 600 r.p.m. for 60 s, followed by pre-annealing at 130 °C for 10 min. After spin-coating at 600 r.p.m. for 20 s, the wet P3HT:ICBA blend film is subjected to solvent-annealing for ≃30 min, followed by post annealing at 150 °C for 10 min.

### PJ device preparation

The PJ device is fabricated by floating a P3HT film on a PCBM film. PCBM and P3HT are both dissolved in chlorobenzene to form a 20-mg ml^−1^ solution. The PCBM film is spin-coated on a ZnO nanocrystal^51^,^52^-modified pre-patterned ITO substrate, followed by thermal annealing at 130 °C for 2 min. The P3HT film is spin-coated on bare glass and, following thermal annealing at 130 °C for 10 min, the film is suspended over a hydrofluoric acid (HF) solution for 1 min and floated in deionized water. After the PCBM-film-coated ZnO/ITO substrate picks up the free-standing P3HT film, the substrate is placed in a vacuum oven (≃1 Pa) for ∼1 h to improve the contact between the bilayer films. Then, a 10-nm MoO_*x*_ layer and a 100-nm Al layer are subsequently evaporated under a pressure of 4 × 10^−4 ^Pa through a shadow mask to define the active area of the devices (0.12 cm^2^) and to form a top electrode.

### Device characterization

The *J–V* characteristics of the devices are recorded using a Keithley 2635A sourcemeter (Keithley Instruments Inc., Cleveland, OH, USA) or an Agilent B1500A-SDA (Agilent Technologies Inc., Santa Clara, CA, USA) under one sun, AM 1.5G irradiation (100 mW cm^−2^) from a solar simulator (Newport 67005, Newport, Irvine, CA, USA). The illumination intensity is calibrated using a reference cell (Oriel 91150V). EQE spectra are measured by a Merlin (Model No. 70104) Digital Lock-in Radiometry System using a 300-W xenon lamp and a 74125 Oriel Cornerstone 260 ¼-m monochromator with order-sorting filters (Newport). The light intensity at different wavelengths is modulated through an aperture and calibrated using a 70714 UV-enhanced Si photodiode (Newport). All of the measurements are performed under ambient atmosphere at room temperature.

### Device cross-section preparation

The device cross-section is fabricated using an Ilion^+^ 693 System (Gatan Inc.). A freshly mechanically cleaved device is mounted into the vacuum chamber (6.4 × 10^−5 ^ Torr) and cooled using liquid nitrogen. Then, the device is milled by argon ions using a beam voltage of 5 keV and a beam current of 10 μA for ≃2 h. Four to five cross-section samples are prepared for each device under study. The *J–V* curves and parameter statistics before and after cross-section preparation are shown in Supplementary Figs 11–13 and Supplementary Tables 1–3.

A control experiment was carried out to examine the ion-beam damage in different materials. As shown in Supplementary Fig. 14, P3HT, PCBM and P3HT:PCBM BHJ thin-film samples are subjected to different time of ion milling, and then imaged with SKPM. The general trend is that the longer ion-milling time, the more material was etched away, and the more significantly the SP changed. The reversal of the SP change as the film became almost completely etched is probably due to the influence of the substrates. These results indicate that the polymer materials are indeed damaged under Ar^+^ ion beam, but freshly exposed surfaces (the shortest time) exhibit similar SP changes compared with respective pristine films (∼50, 30 and 60 mV for P3HT, PCBM and P3HT:PCBM thin films, respectively); therefore, the relative SP difference values are reliable. Consider the ion-milling configuration in cross-section preparation (Supplementary Fig. 15), the cross-section surface finally exposed for SKPM measurements can be considered fresh surface since majority of etching and damage happens at the frontier of ion beam rather than the sideway.

### Device cross-section characterization

The morphology of the device cross-section is characterized using FEI Quanta 400 FEG SEM (FEI Corp., Hillsboro, OR, USA). The SKPM SP profile measurements of device cross-sections are carried out using a Park XE-120 AFM (Park Systems Corp., Suwon, Korea) using Ti/Pt-coated conducting tips (PPP-EFM, Nanosensor, Neuchatel, Switzerland) with a resonance frequency of ∼80 kHz and a spring constant of ∼0.6 N m^−1^. A two-pass scan AM mode SKPM is used to measure the SP under an environment with relative humidity below 20%. During the first pass, standard AC mode imaging (typical tip oscillation amplitude 20 nm) is performed to acquire the topography and phase signal of the sample; in the second pass, the tip is lifted up by a certain height (typically 10 nm) and scanned on the basis of the topography line obtained from the first pass. An AC voltage (3 V in amplitude and 10 kHz in frequency) is applied to actuate the cantilever, and the DC voltage applied to the tip that nullifies the tip–sample interaction is collected as the SP signal. The device-wiring configuration during cross-section characterization is shown in Fig. 1b. The Al electrode is grounded. The ITO electrode is directly connected with the Al electrode in short-circuit; open-circuit conditions were simulated by disconnecting the wire between two electrodes. A full-spectrum optical fibre is used to transmit AM 1.5 G solar simulator light to illuminate the device from the ITO glass side. Device forward or reverse bias voltage was applied via a tuneable voltage source between the electrodes.

## Additional information

**How to cite this article:** Chen, Q. *et al.* Quantitative *operando* visualization of the energy band depth profile in solar cells. *Nat. Commun.* 6:7745 doi: 10.1038/ncomms8745 (2015).

## Supplementary Material

Supplementary Information Supplementary Figures 1-15, Supplementary Tables 1-3, Supplementary Note 1 and Supplementary Reference

## Figures and Tables

**Table 1:** Device parameters of P3HT:PCBM and P3HT:ICBA BHJ devices before and after cross-section preparation and SKPM measurement.

**Active layer**	**Device**	***V***_**oc**_ **(V)**	***J***_**sc**_ **(mA cm**^−**2**^**)**	**FF**	**PCE (%)**
P3HT:PCBM	Original	0.61	9.17	0.62	3.47
	Cross-sectioned	0.62	8.53	0.60	3.17
	After SKPM	0.62	7.50	0.57	2.65
P3HT:ICBA	Original	0.84	9.41	0.62	4.90
	Cross-sectioned	0.84	8.96	0.59	4.44
	After SKPM	0.84	7.79	0.56	3.66

BHJ, bulk heterojunction; FF, fill factor; ICBA, indene-C_60_ bisadduct; PCBM, [6,6]-phenyl-C_61_-butyric acid methyl ester; P3HT, poly(3-hexylthiophene); SKPM, Scanning Kelvin probe microscopy.
