# Supplementary material for: Quantitative operando visualization of the energy band depth profile in solar cells
Source: Nat Commun. 2015 Jul 13;6:7745. doi: 10.1038/ncomms8745 (PMC4510960; doi:10.1038/ncomms8745)
Supplement: Supplementary Information — Supplementary Figures 1-15, Supplementary Tables 1-3, Supplementary Note 1 and Supplementary Reference [file ncomms8745-s1.pdf]

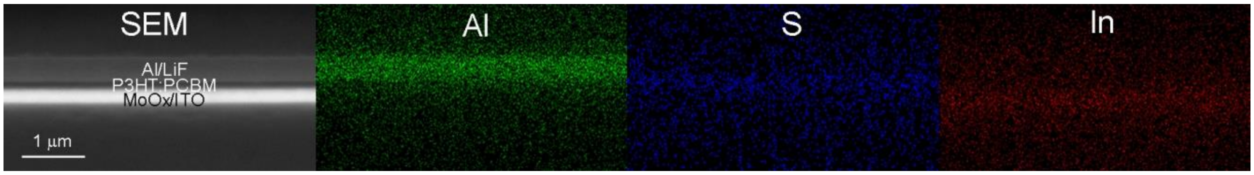

Supplementary Figure 1. SEM micrograph of the ion milled OPV cross section and the corresponding EDX mapping of Al, S, and In elements.

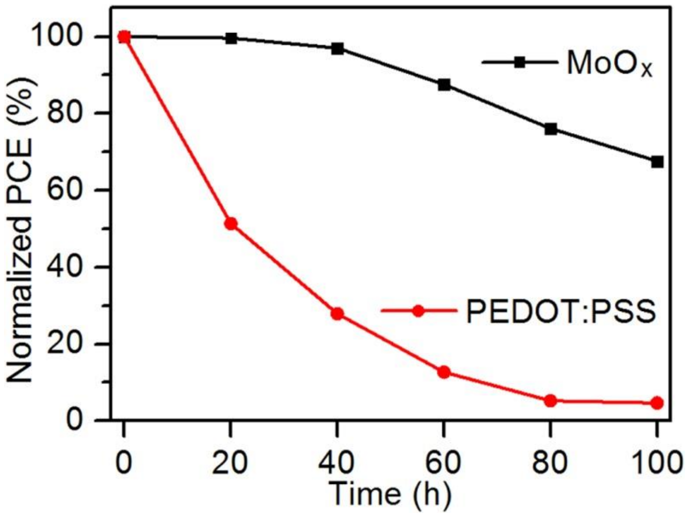

Supplementary Figure 2. The stability of devices with the structures of ITO/MoO_x/P3HT:PCBM/LiF/Al and ITO/PEDOT:PSS/P3HT:PCBM/LiF/Al within 100 h.

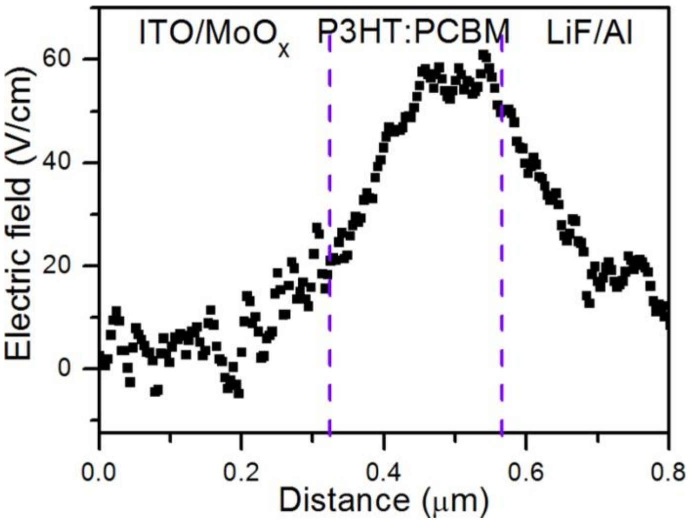

Supplementary Figure 3. Electric field across a P3HT:PCBM BHJ device in dark state, obtained via taking the derivative of the surface potential profile in Figure 2g.

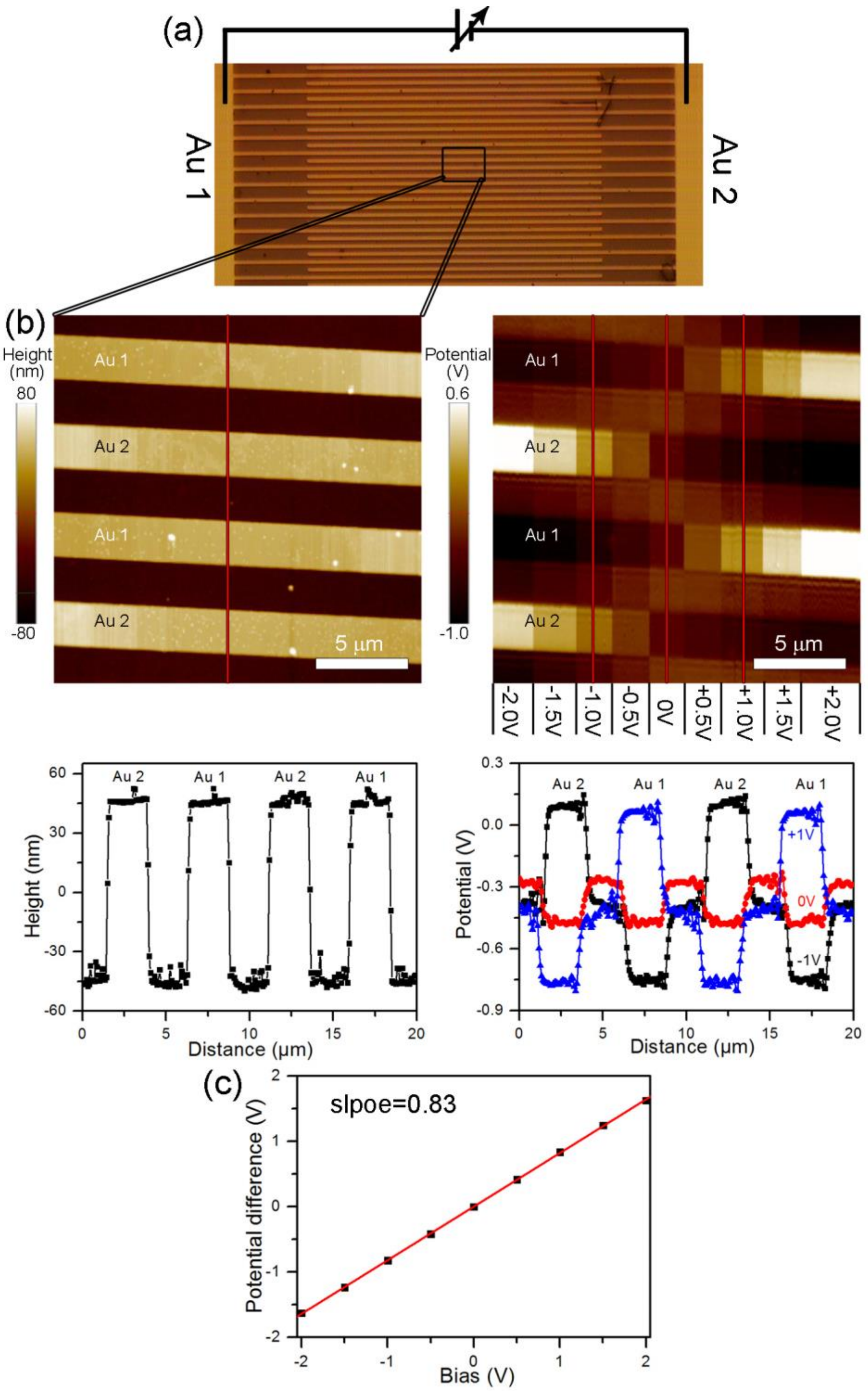

Supplementary Figure 4. (a) optical microscopy image of a pair of interdigitating Au electrodes (electrode width 2.5 µm, spacing between electrodes: 2.5 µm) for calibration of quantitative potentiometry; (b) topography and surface potential image of the cross-finger Au electrodes. The height profile and potential profiles at -1.0 V, 0 V and +1.0 V bias voltage, (c) Linear correlation between the measured potential difference and the applied bias (vertical error bar for each data point is about 20 meV).

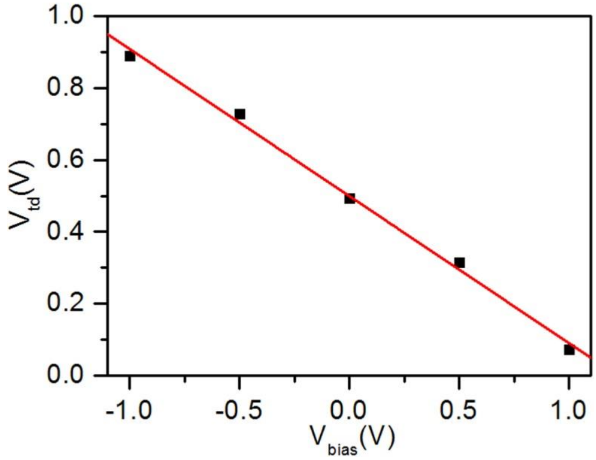

Supplementary Figure 5. The plot of surface potential drop from cathode to anode (V_{td}) versus applied bias voltage (V_{bias}) for the ITO/MoO_x/P3HT:PCBM/LiF/Al device.

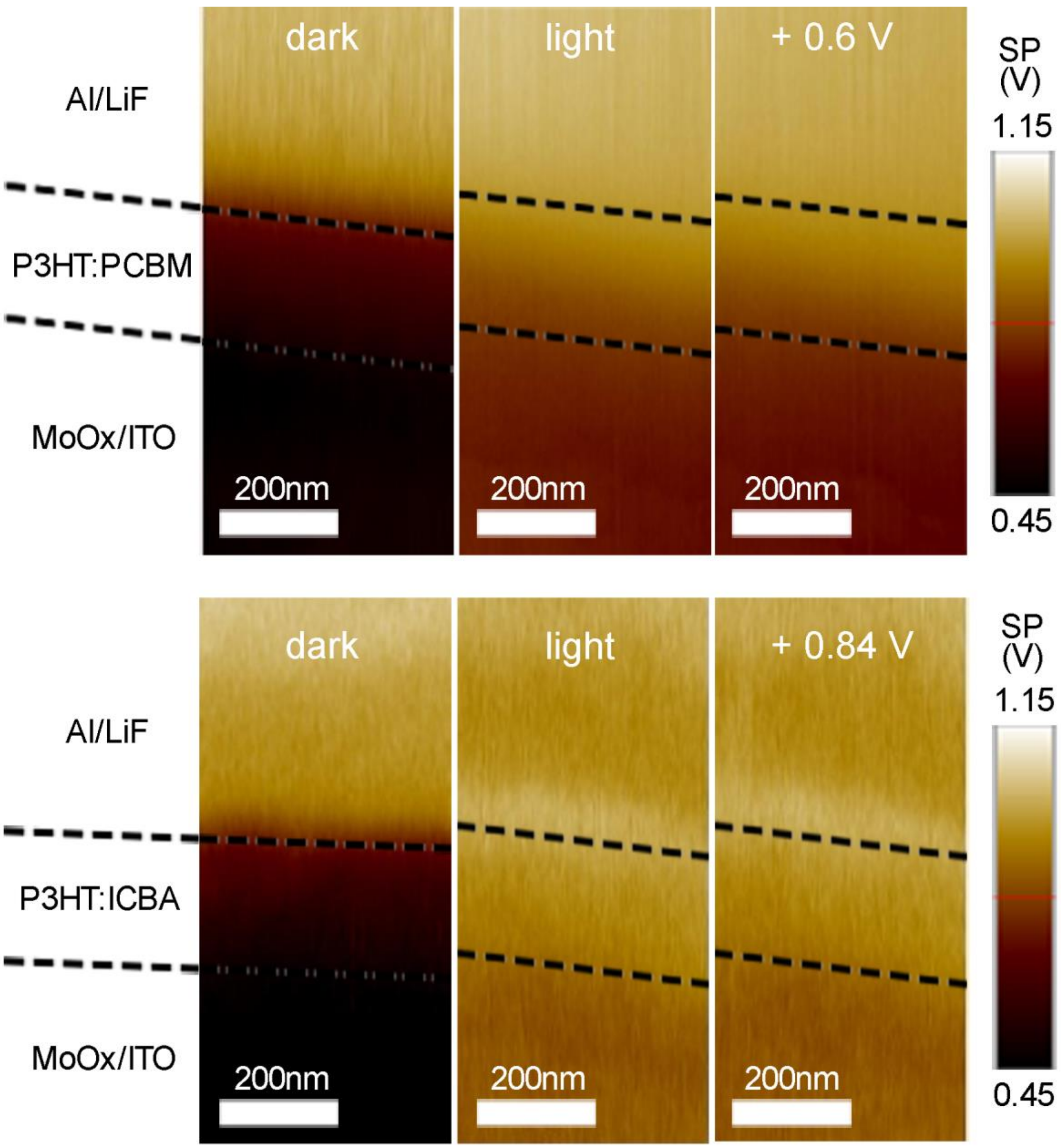

Supplementary Figure 6 SP images of P3HT:PCBM and P3HT:ICBA BHJ devices in dark, under illumination and under a specific bias voltage.

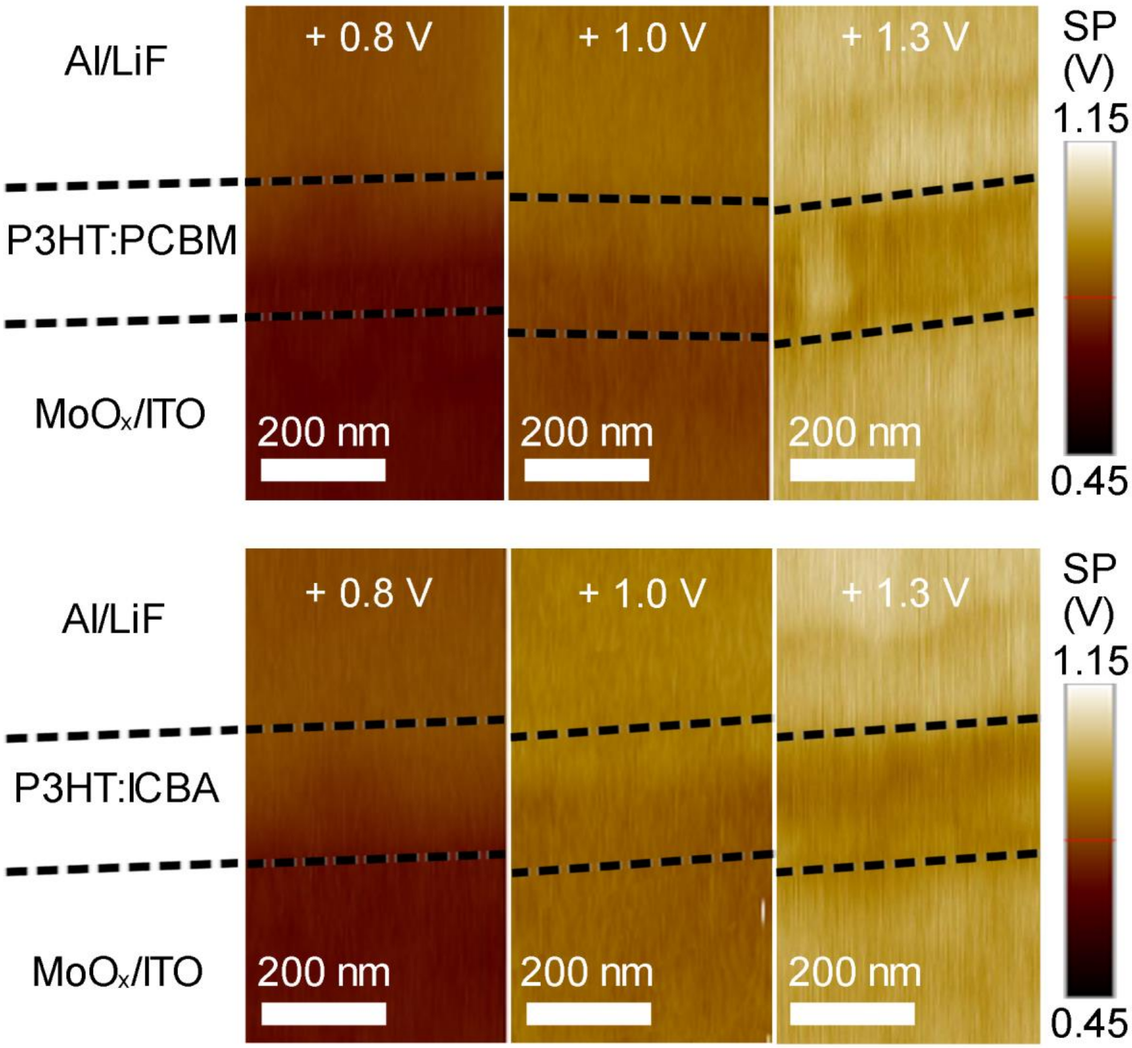

Supplementary Figure 7. SP images of P3HT:PCBM and P3HT:ICBA BHJ devices under +0.8 V, +1.0 V and +1.3 V bias voltages.

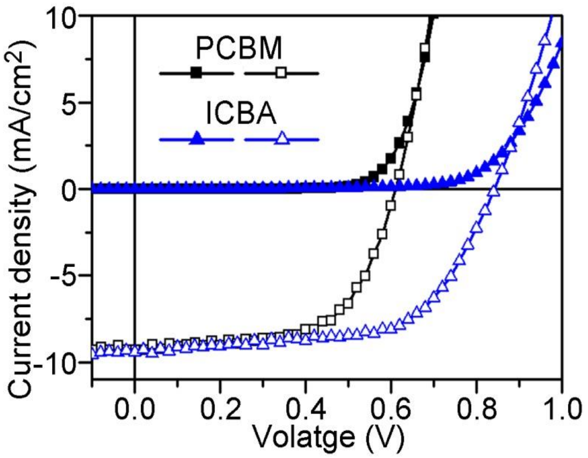

Supplementary Figure 8. *J*–*V* characteristics of the P3HT:PCBM and P3HT:ICBA BHJ OPV devices with conventional configuration ITO/MoO_x/BHJ/LiF/Al in dark and under AM 1.5G illumination.

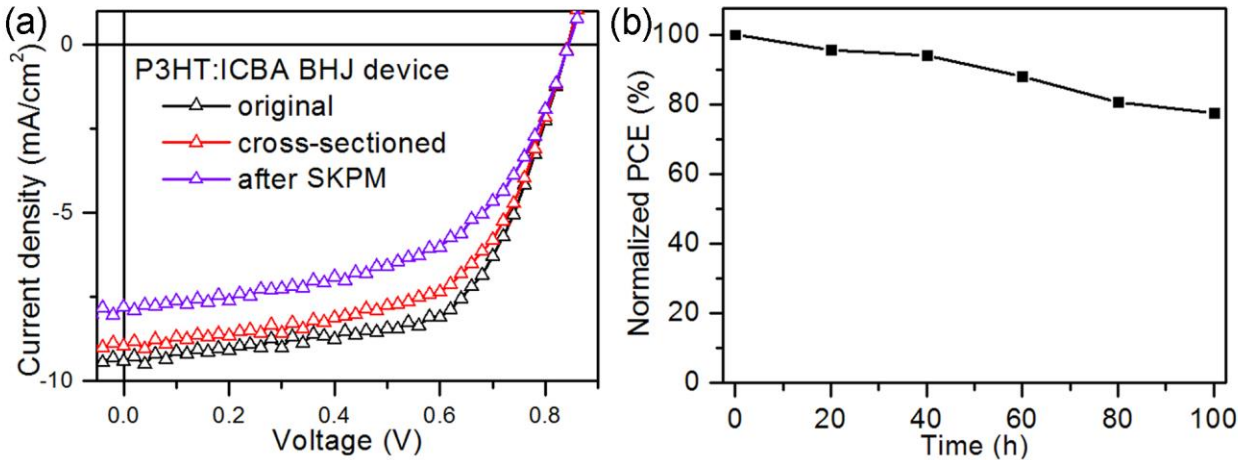

Supplementary Figure 9. (a) Current density versus voltage (*J*–*V*) characteristics of the OPV device with conventional configuration ITO/MoO_x/P3HT:ICBA/LiF/Al under AM 1.5G illumination before (black) and after (red) cross section preparation, and after SKPM measurement (violet). The extracted device parameters are listed in Table 1. (b) The stability of the P3HT:ICBA device within 100 h.

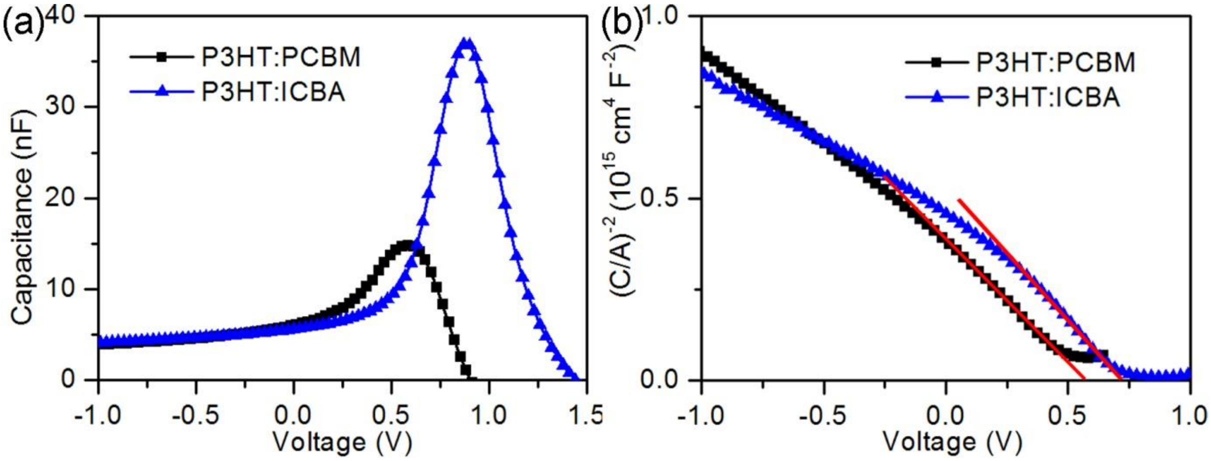

Supplementary Figure 10. Capacitance versus voltage (C-V) curves (a) and the corresponding Mott-Schottky plots (b) of P3HT:PCBM (solid black square) and P3HT:ICBA (solid blue triangle) BHJ devices in dark with device stacking: ITO/MoO_x/active layer/LiF/Al.

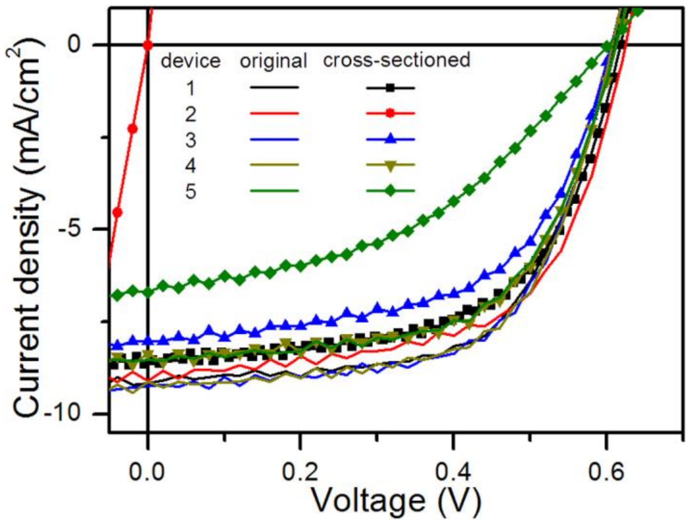

Supplementary Figure 11. J-V characteristics of P3HT:PCBM BHJ devices before and after cross section preparation.

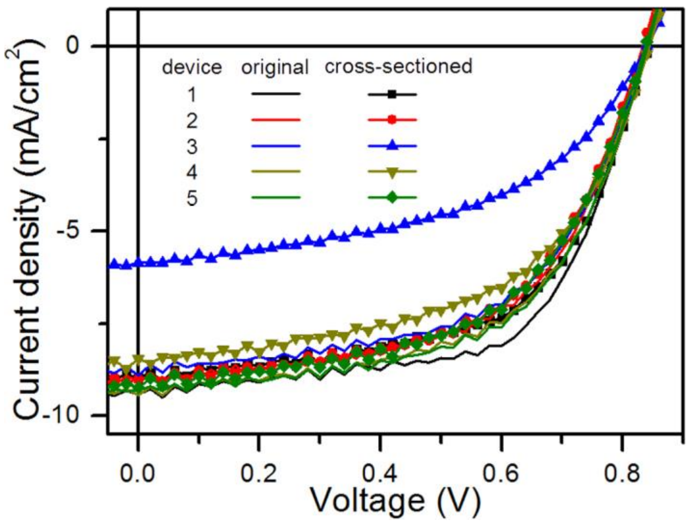

Supplementary Figure 12. J-V characteristics of P3HT:ICBA BHJ devices before and after cross section preparation.

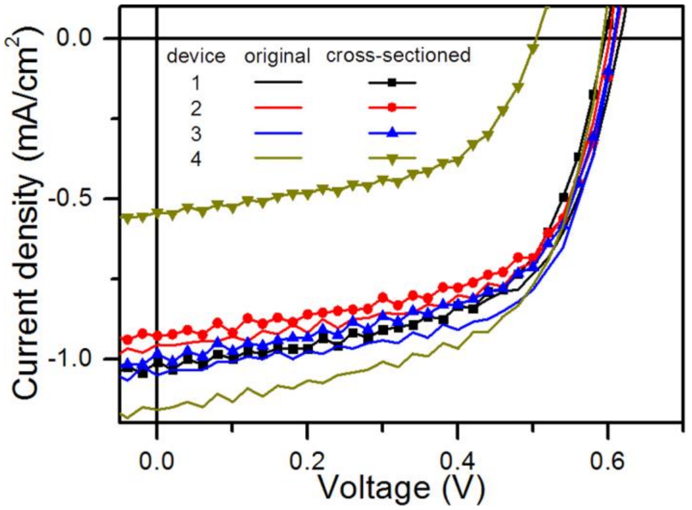

Supplementary Figure 13. J-V characteristics of P3HT/PCBM PJ devices before and after cross section preparation.

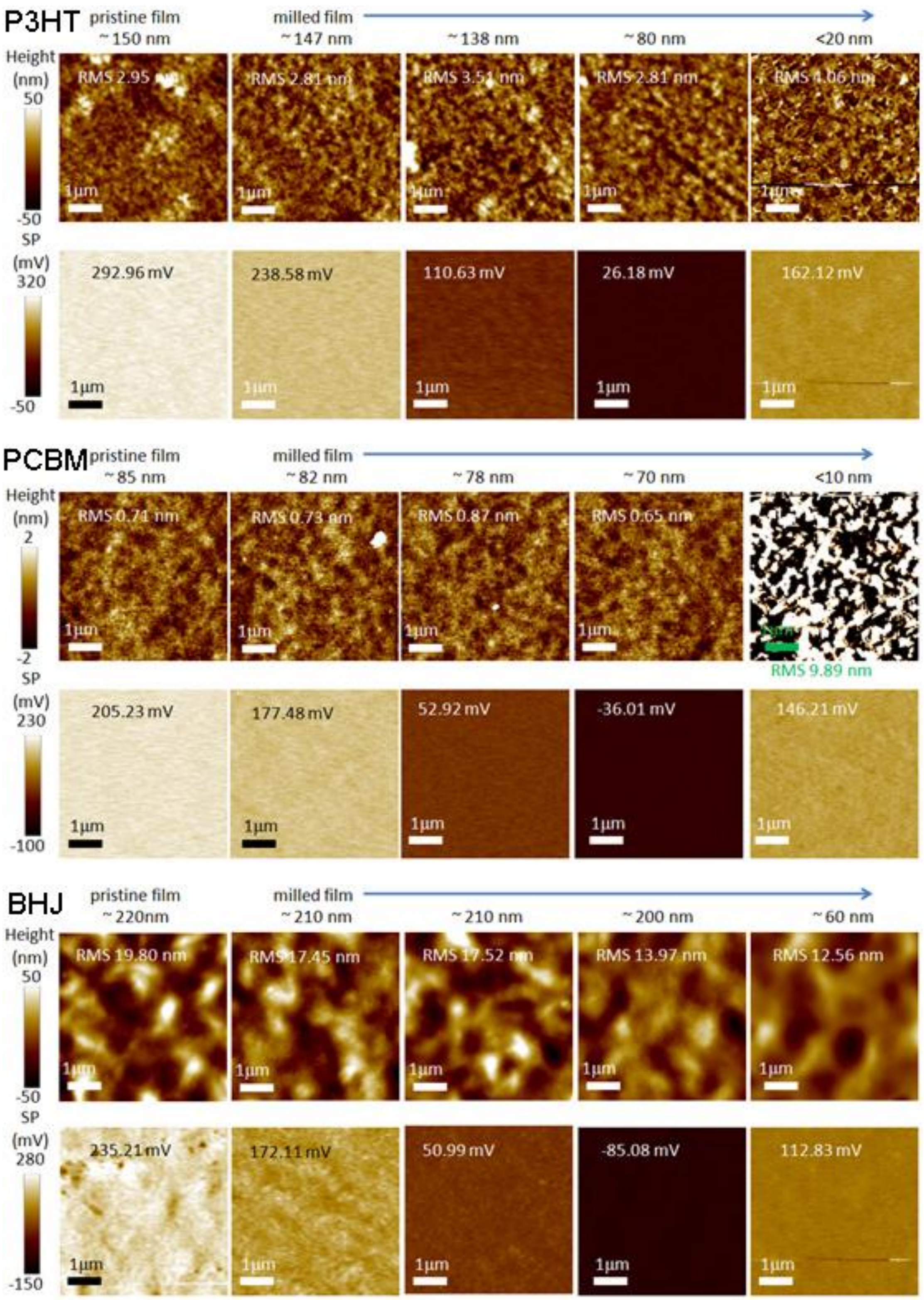

Supplementary Figure 14. The SP change upon ion beam etching in P3HT, PCBM and P3HT:PCBM BHJ thin films. All materials were dissolved in o-dichlorobenzene, the same solvent in device preparation, for spin-casting. The thickness of the film after etching was very coarsely controlled by the etching time.

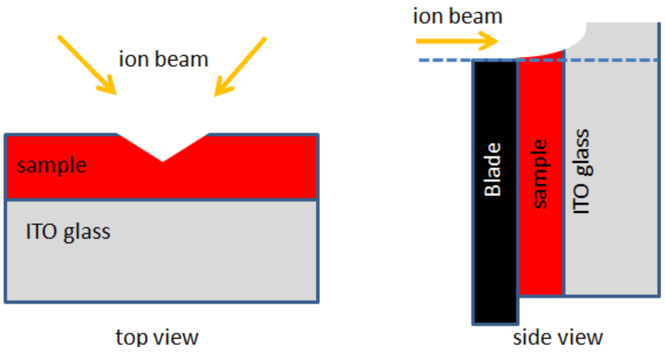

Supplementary Figure 15. Schematic illustration of the ion beam etching configuration for the thin-film samples above and for the OPV device cross section preparation.

Supplementary Table 1 P3HT:PCBM BHJ device performance statistics before and after cross section preparation

P3HT:PCBM	device	status	V_{oc} (V)	J_{sc} (mA cm^{-2})	FF	PCE (%)
1	original		0.61	9.17	0.62	3.47
	cross-sectioned	*operando*	0.62	8.53	0.60	3.17
2	original		0.61	9.08	0.62	3.43
	cross-sectioned	short-circuit	--	--	--	--
3	original		0.61	9.25	0.63	3.55
	cross-sectioned	*operando*	0.61	8.02	0.58	2.84
4	original		0.61	9.13	0.62	3.45
	cross-sectioned	*operando*	0.61	8.36	0.61	3.11
5	original		0.61	8.55	0.60	3.13
	cross-sectioned	deteriorate	0.60	6.70	0.43	1.73

Supplementary Table 2 P3HT:ICBA BHJ device performance statistics before and after cross section preparation

P3HT:ICBA	device	status	V_{oc} (V)	J_{sc} (mA cm^{-2})	FF	PCE (%)
1	original		0.84	9.41	0.62	4.90
	cross-sectioned	*operando*	0.84	8.96	0.59	4.44
2	original		0.84	9.08	0.57	4.35
	cross-sectioned	*operando*	0.84	8.99	0.57	4.30
3	original		0.84	8.89	0.57	4.26
	cross-sectioned	deteriorate	0.84	5.85	0.49	2.41
4	original		0.84	9.43	0.57	4.52
	cross-sectioned	deteriorate	0.84	8.77	0.50	3.68
5	original		0.84	9.31	0.59	4.61
	cross-sectioned	*operando*	0.84	9.22	0.56	4.34

Supplementary Table 3 P3HT/PCBM PJ device performance statistics before and after cross section preparation

PCBM/P3HT	device	status	V_{oc} (V)	J_{sc} (mA cm^{-2})	FF	PCE (%)
1	original		0.61	1.01	0.61	0.38
	cross-sectioned	*operando*	0.60	1.01	0.60	0.36
2	original		0.60	0.95	0.61	0.35
	cross-sectioned	*operando*	0.60	0.93	0.60	0.33
3	original		0.61	1.01	0.62	0.38
	cross-sectioned	*operando*	0.61	0.98	0.59	0.35
4	original		0.59	1.13	0.57	0.36
	cross-sectioned	deteriorate	0.51	1.09	0.56	0.31

Supplementary Note 1. Mott-Schottky analysis

Mott-Schottky analysis of capacitance versus voltage (C-V) curve, which assumes a constant doping level profile within the organic material:

$$C^{-2} = \frac{2(V_{fb} - V)}{q\varepsilon_0\varepsilon N} \qquad \text{(Equation 1)}$$

where V_{fb} is the flat-band potential, V is the applied voltage, q is the elementary charge, ε_0 is the permittivity of the vacuum, ε is the relative dielectric constant (assuming ε~3 for P3HT based BHJ)[1], and N is the concentration of doping level. A linear relationship is observed in the C^{-2} – V plot when the applied voltage modulates the depletion layer width. According to Equation 1, V_{fb} can be derived from the intercept of linear fitting curve, and N is calculated from the slope of curve.

Supplementary Reference

1 Guerrero, A. *et al.* How the Charge-Neutrality Level of Interface States Controls Energy Level Alignment in Cathode Contacts of Organic Bulk-Heterojunction Solar Cells. *ACS Nano* **6,** 3453-3460 (2012).
